# Factors Associated with Non-Compliance with Healthcare Accreditation in Saudi Arabia: A Systematic Review and Meta-Analysis

**DOI:** 10.3390/healthcare13060580

**Published:** 2025-03-07

**Authors:** Khalid Saad Alkhurayji, Abdulmunim Alsuhaimi, Hesham Alangari, Saja A. Alrayes, Arwa Alumran

**Affiliations:** 1 Research, Statistics, and Information Department, Saudi Central Board for Accreditation of Healthcare Institutions, Riyadh 12264, Saudi Arabia; 2 Executive Department of Standards, Saudi Central Board for Accreditation of Healthcare Institutions, Riyadh 12264, Saudi Arabia; aalsuhaimi@cbahi.gov.sa; 3 Development of Standards and Evaluation System Section, Saudi Central Board for Accreditation of Healthcare Institutions, Riyadh 12264, Saudi Arabia; halangari@cbahi.gov.sa; 4 Health Information Management and Technology Department, College of Public Health, Imam Abdulrahman Bin Faisal University, Dammam 31451, Saudi Arabia; salrayes@iau.edu.sa (S.A.A.); aalumran@iau.edu.sa (A.A.)

**Keywords:** global health, delivery of healthcare, health services administration, quality of healthcare, public health

## Abstract

**Background/Objectives**: Compliance with healthcare standards is an absolute must for every healthcare organization seeking accreditation. Several factors were found to affect compliance, and in Saudi Arabia, certain standards were observed for non-compliance. Therefore, this systematic review and meta-analysis seeks to identify the factors associated with non-compliance with healthcare accreditation in Saudi Arabia. **Methods**: This study adheres to the Preferred Reporting Items for Systematic Review and Meta-Analysis (PRISMA) guidelines. The population, intervention, comparison, and outcome (PICO) model was used to refine the research question. The Peer Review of Electronic Search Strategies (Press) guidelines were used to improve the search strategy. The databases used for the search were PubMed, Web of Science, Scopus, and Google Scholar. The dates searched were from 1 January 2000 to 1 November 2024. We used a data extraction form for study characteristics and outcome data, which was piloted on five studies in this review. The risk of bias was assessed by using the Joanna Briggs Institute (JBI) tool and the Mixed Methods Appraisal Tool (MMAT). The analysis was carried out using the Fisher r-to-z transformed correlation coefficient as the outcome measure. A random-effects model was fitted to the data. **Results**: A total of ten studies were included for qualitative synthesis and five for quantitative synthesis. Several factors were observed for non-compliance, including insufficient training organization hurdles, a lack of implementation strategies, and the attitudes of healthcare providers. The estimated average correlation coefficient based on the random-effects model was 0.2568 (95% CI: −0.1190 to 0.6326). **Conclusions**: The dimension of quality in healthcare through pooled correlations from various studies highlighted a weak association among these dimensions.

## 1. Introduction

Compliance with healthcare standards is an absolute must for every healthcare organization seeking accreditation [1]. Healthcare institutions strive for certification to promote higher quality and patient safety [2]. However, non-compliance with national and international standards remains a significant concern, and healthcare institutions may fail to comply due to a variety of factors [3].

Several factors were found to affect compliance, for instance, insufficient training, lack of equipment, and hospital infrastructure [4,5,6]. Non-compliance could cause several issues related to patient care and healthcare service outcomes, which could impact the overall healthcare provision [7]. According to previous systematic reviews, accreditation can improve the quality of healthcare, and accreditation continues to be accepted internationally. However, factors that affect compliance are required to be assessed to improve the overall healthcare system for the future application of the accreditation process and the development of national and international standards [8].

Previous investigations in terms of reviews illustrate that the factors influencing non-compliance with healthcare accreditation among hospitals include the hospital’s ability to comply, linking to funding mechanisms, the mandatory versus voluntary nature of accreditation, staff engagement and communication, leadership and staff training, increased staff workload, the integration and utilization of information, the adoption of accreditation standards, and the impacts of hospital accreditation [2,9].

In Saudi Arabia, certain standards were observed for non-compliance, such as failure to adhere to infection control protocols, non-compliance with sanitation standards, and not following established patient safety guidelines [10,11,12,13]. However, the Saudi Central Board for Healthcare Institutes (CBAHI) oversees the implementation and enforcement of these standards to ensure that healthcare facilities provide safe and effective care [14].

The significance and novelty of this review compared to previous reviews are that this review aims to uncover the factors related to non-compliance with healthcare accreditation in Saudi Arabia through a systematic review and meta-analysis, with an attempt to influence the solutions to enhance compliance and, ultimately, patient safety and quality. Through synthesizing evidence from current research, this examination could provide a thorough understanding of the obstacles affecting healthcare accreditation compliance in Saudi Arabia, emphasizing key areas of non-compliance and feasible ways to solve these challenges. Therefore, non-compliance is a subject that needs to be addressed and analyzed to look for a solution that would help in mitigating and eradicating the non-compliance factors. Therefore, this study aims to answer the following research question: what are the factors associated with non-compliance with healthcare accreditation standards in Saudi Arabia?

## 2. Materials and Methods

This study complies with the guidelines of the Preferred Reporting Items for Systematic Review and Meta-Analysis (PRISMA), as shown in File S1. The population, intervention, comparison, and outcome (PICO) model was used to refine the research question (Table 1). The Peer Review of Electronic Search Strategies (Press) guidelines were used to improve the search strategy, as shown in Supplementary File S4.

### 2.1. Eligibility Criteria

All relevant before–after studies, cross-sectional studies, surveys, or cohort studies pertaining to healthcare facilities in Saudi Arabia were included as long as they contained the post-intervention accreditation. Our primary outcome of interest was factors associated with non-compliance. Restrictions were applied to the publication types. Conference abstracts, theses, articles in the press, and books or book chapters were purposefully removed from the search results. We only included studies in the English language.

**Figure 1:**
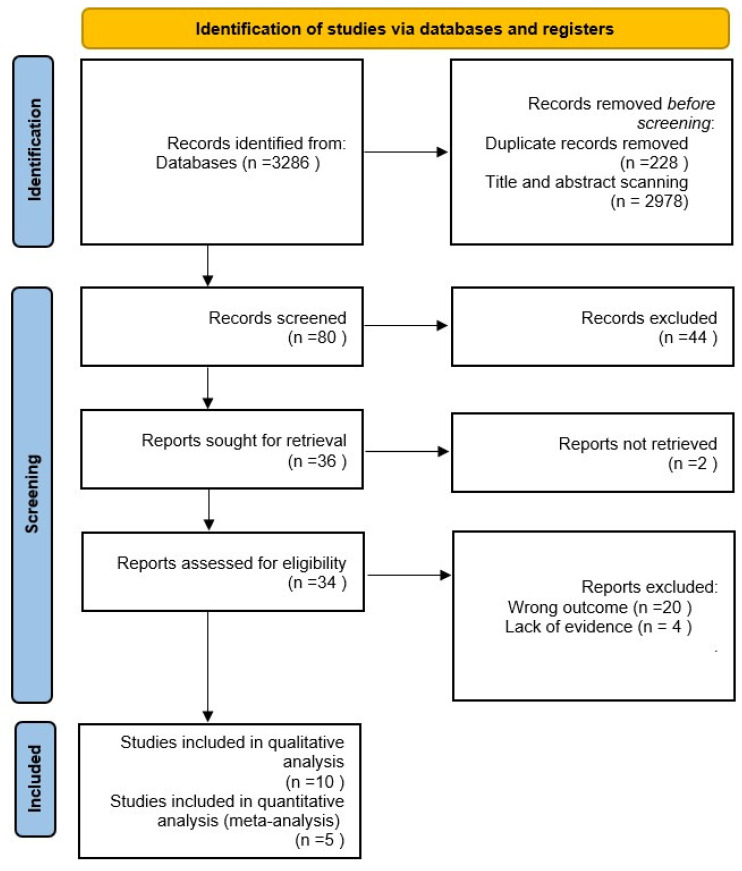
PRISMA flowchart.

Articles were screened by title and abstract independently by the authors (K.S.A., H.A.). For articles eligible after screening, the full texts were retrieved by K.S.A. and H.A., and were reviewed by K.S.A. and H.A. The citation search was screened by K.S.A. and H.A. Discrepancies were resolved by referring to a third author A.A. (Abdulmunim Alsuhaimi). The selection process was recorded in sufficient detail to complete the Preferred Reporting Items for Systematic Reviews and Meta-Analysis (PRISMA) flow diagram (Figure 1).

### 2.2. Search Strategy

The components described below were included in the search string synonyms and search filters. The search strings were designed by an information specialist. Additionally, the search was peer-reviewed by a doctor of public health. The databases used for the search were PubMed, Web of Science, Scopus, and Google Scholar. The dates searched were from 1 January 2000 to 1 November 2024, as shown in Supplementary File S2. To supplement the database search, we manually checked the reference lists of the included studies and contacted experts.

### 2.3. Data Extraction

We used a data extraction form for the study characteristics and outcome data, which was piloted on five studies in the review. The study authors (K.S.A., H.A.) extracted the following data from the included studies:

(a). Types: before–after studies, cross-sectional studies, surveys, and cohort studies.
(b). Methods: study authors, year, study design, setting, and region.
(c). Participants: number of participants.
(d). Interventions: accreditation.
(e). Outcomes (study variables): factors associated with non-compliance.

### 2.4. Risk of Bias Assessment

Two authors assessed the risk of bias for each study using the JBI tool and MMAT [15,16].

### 2.5. Data Synthesis

Data will be presented in tabulation as well as in graphics such as forest plots, with the majority of our data being presented narratively.

### 2.6. Missing Data

Where data were missing, the study investigators or sponsors were contacted.

### 2.7. Meta-Correlation Analysis

From the five studies included in the meta-analysis, the authors identified the variables associated with non-compliance, which included insufficient training, continuous training, lack of availability of personal protective equipment, ownership of the hospital, and bed capacity. These variables were interrelated and collectively impacted the robustness of health infrastructure [17]. The remaining five studies were removed from the meta-analysis due to a lack of necessary elements for computing the correlation coefficient and having a research design that did not include quantitative measurement. We performed a meta-analysis with the use of Jamovi software (with major meta-analysis packages (version 2.4.14) [18]. The computing and converging of the effect size r (correlation coefficient) was executed through psychometric website [19]. The χ^2^ and z test statistics from the hypothesis tests could be used to compute d and r [20]. Peterson and Brown [21] suggest a procedure for converting standardized β weights to r, if the β weights range between −0.5 and 0.5. r can then be used directly as an effect size or converted into d or other metrics. Nonetheless, the transformation for r can be conducted through the transformation of Cohen’s d through the odd ratio, and the f value from the ANOVA test, in addition to the mean and standard deviation from the two groups, can be defined as Cohen’s d, which then can be computed to r. Moreover, the test statistics can be transformed in effect size r, such as Wilcoxon, Mann–Whitney U, or Chi-square [20,22,23].

For the post-computing of r, each variable was weighed using Fisher’s z-transformation to the raw correlation coefficients to stabilize their variance [24]. After transforming the correlation coefficients into z-scores, they were weighted, and then the results were transformed back into correlation coefficients for reporting [25]. All studies were similar in terms of their design and the relationships they measured between the same types of variables, which made the comparison of the correlations statistically sound. Furthermore, the use of Fisher’s z-transformation further ensured that the distribution of correlation coefficients was approximately normal, allowing us to perform a valid comparison even when the studies differed in sample size [23,26].

The result metric of the analysis was the Fisher r-to-z transformed correlation coefficient. The data were analyzed using a random-effects model. The restricted maximum-likelihood estimator was used to estimate the level of heterogeneity (tau^2^) [27]. In addition, I^2^ statistics, tau^2^, and the Q-test for heterogeneity were estimated [28]. If heterogeneity was discovered (e.g., tau^2^ > 0, regardless of Q-test findings), a prediction interval for true outcomes was presented. Studentized residuals and Cook’s distances were used to determine if the studies were outliers or influential in the context of the model. Studies having a studentized residual greater than 100 × (1 − 0.05/(2 × k)) the percentile of a typical normal distribution were regarded as potential outliers (using a Bonferroni adjustment with two-sided alpha = 0.05 for k studies included in the meta-analysis). Studies with a Cook’s distance greater than the median plus six times the interquartile range of the Cook’s distances were considered influential. To assess funnel plot asymmetry, the rank correlation test and the regression test were employed, with the standard errors of the observed results as predictors.

## 3. Results

### 3.1. Characteristics of Studies Included

This review provides a detailed overview of the process for identifying the studies from various databases and search engines. The identification phase revealed 3286 records for the relevant studies. Following the removal of 228 studies for duplicated records, 2978 records were scanned by title and abstract, indicating a systematic narrowing down of the studies based on initial relevance. The screening phase involved 80 records, which were screened against our inclusion and exclusion criteria, showing a rigorous filtering process. The second stage revealed 44 records that were excluded. Additionally, due to non-retrieval conditions, two studies were excluded. With that, thirty-four studies were assessed by full-text screening, resulting in the removal of twenty studies, in addition to four reports that were excluded due to a lack of evidence and twenty for having the wrong outcome. This resulted in a final total of ten studies for qualitative synthesis and five for quantitative synthesis, indicating that these studies provided sufficient data to contribute to the statistical synthesis and evaluation of results.

### 3.2. Study Overview

Table 2 summarizes the studies completed between 2009 and 2022 in various regions of Saudi Arabia on patient safety and healthcare quality. The study efforts demonstrated that the determinants of non-compliance are related to the workplace and healthcare contexts. Furthermore, these papers demonstrated varied sample sizes and research designs. Nonetheless, this provided a broad range of data collected over time. The regions featured in these studies were Qassim, Asser, Riyadh, Makkah, and Dammam, which reflected diverse locations and settings.

### 3.3. Research Design Insights

This review found several research designs, including cross-sectional, cohort, and mixed methods, that presented a comprehensive understanding of compliance factors from different angles.

### 3.4. Critical Appraisal of Included Studies

The critical appraisal of the included research revealed that the majority of studies had a high level of quality ranging from 75% to 100%. However, two studies had low percentage scores of 62.5% for a cross-sectional study and 54.45% for cohort research, as seen in File S3.

### 3.5. Compliance Rates

The compliance rate was reported to be high in some studies, with a mean score of 4.64, which was observed in Shahin, Alshammari, and Alabed’s [29] study. However, it was observed that it did not exceed a 20% compliance rate despite participants having adequate knowledge [30].

### 3.6. Factors for Non-Compliance

Several factors were observed for non-compliance, including insufficient training, organizational hurdles, a lack of implementation strategies, and the attitudes of healthcare providers [29,31,32,33]. Additionally, some studies reported a lack of personal protective equipment and perceived non-importance, as seen in a 2015 study [34,35]. Moreover, another study highlighted that there being no prioritization of documentation was reported to impact the compliance rate, which has been observed between private (77%) and public hospitals (66%). Larger hospitals were observed to have more compliance compared to smaller ones (81% vs. 66%) [36]. Moreover, healthcare providers were reported in specialized hospitals to demonstrate a high level of knowledge of patient identification protocols (87.1%) but they had a low implementation rate (18%) [33]. Some healthcare professionals did not prioritize documentation, impacting compliance. The healthcare providers’ attitudes were frequently mentioned as significant factors in non-compliance [34]. Training was among the major factors for the compliance rate among healthcare providers. In fact, this suggests that ongoing professional development is essential for improving healthcare compliance [30].

### 3.7. Distribution Across Dimensions

Each dimension found various study contributions to gain a better understanding, with the total number of studies varying by dimension. The majority of studies were distributed equally in the safe, effective, and efficient factors; however, one study was related to patient-centered factors, and additionally, no studies were found in the dimension of time or in all domains together, as shown in Table 3.

**Table 2:** Key characteristics of included studies.

Reference	Study Year	Sample Size	Research Design	Region	Type of Facility	r	Factor for Non-Compliance	Summary Outcome	Domain	CA
[29]	2020	147	Cross-sectional	Qassim	Hospital	0.49	Insufficient training	The overall compliance with International Patient Safety Goals (IPSGs) was very high, with a mean score of 4.64.	Efficient and Effective	87.5
[30]	2018	82	Cross-sectional	Makkah	Primary Healthcare Centers	−0.110	Continuous Training	While 78% of DHCPs were classified as having adequate knowledge, only 19.5% demonstrated adequate compliance.	Efficient and Effective	75.0
[35]	2018	342	Cross-sectional	Aseer	Public hospital	0.858	Lack of availability of personal protective equipment	Both a high awareness of necessary measures and significant barriers to compliance were present, such as discomfort with protective clothing and workload during emergencies.	Safe	75.0
[37]	2016, 2017, and 2018,	437	Cross-sectional	Multiple regions	Both private and public hospitals	−0.152	Ownership of hospital	Private hospitals had a significantly higher compliance rate (77%) compared to public hospitals (66%). Additionally, hospitals with a bed capacity of more than 200 had a higher compliance rate than those with 200 or fewer beds (81% vs. 66%).	Equitable	100.0
[36]	2018	318	Cross-sectional	Multiple regions	Hospital	0.1733	Bed capacity	The overall hand hygiene (HH) compliance rate was found to be 70%. Nurses had the highest compliance at 73.3%, followed by doctors at 67.3%, and other allied healthcare workers (HCWs) at 66.1%.	Efficient and Effective	75.0
[34]	2015	805	Cross-sectional	Riyadh	Hospital	-	Perceived non-importance of certain documentation by pharmacists	The number of reports was high. However, there was a need for more determination to ensure the completeness of the medication error reporting system, which could be improved through the development of an electronic reporting system and increased program awareness.	Safe	62.5
[32]	2022	657	Mixed methods	Riyadh	Hospital	-	Organizational hurdles	Healthcare provider non-compliance with the (PAMP) recommendations was primarily due to organizational obstacles, the lack of an implementation strategy, and the attitudes and beliefs of healthcare providers.	Safe, Efficient, and Effective	80.0
[31]	2015–2021	90,007	Cohort	Dammam	Hospital	-	Absence of a strategy for protocol implementation	The random-forest classifier was identified as the best-fit model for predicting documentation compliance. This model helped to identify physicians at risk of non-compliance with EHR documentation.	Safe	54.54
[33]	2015–2017	791	Cross-sectional	Riyadh	Specialized hospital	-	Attitudes and beliefs of healthcare providers	Healthcare providers showed high knowledge (87.1%) of patient identification protocols but poor implementation, with only 18% following proper identification practices during audits.	Patient-centered	100
[38]	2009	657	Mixed methods	Riyadh	Hospital	-	Organizational factors (e.g., staff and bed shortages, high patient volume during winter); Attitudes and beliefs of healthcare providers	The study found that organizational barriers, the lack of an implementation strategy for the Pediatric Asthma Management Protocol (PAMP), and the attitudes and beliefs of healthcare providers were the main factors contributing to non-compliance with the PAMP recommendations	Safe, Efficient, and Effective, Patient-centered	100

r = correlation coefficient; CA = critical appraisal; values of 50% or below indicate a low-quality article, values between 50% and 69% are considered average quality, and 70% represents high-quality articles.

### 3.8. Meta Correlation Analysis

A total of k = 5 studies were included in the analysis. The observed correlation coefficients ranged from −0.1522 to 0.8583, with the majority of estimates being positive (60%). The estimated average correlation coefficient based on the random-effects model was 0.2568 (95% CI: −0.1190 to 0.6326). Therefore, the average outcome did not differ significantly from zero (z = 1.3392, *p* = 0.1805). According to the Q-test, the true outcomes appeared to be heterogeneous (Q(4) = 612.7635, *p* < 0.0001, tau^2^ = 0.1797, I^2^ = 98.8908%). A 95% prediction interval for the true outcomes was given by −0.6550 to 1.1686. Hence, although the average outcome was estimated to be positive, in some studies the true outcome may in fact have been negative. An examination of the studentized residuals revealed that none of the studies had a value larger than ± 2.5758, and hence there was no indication of outliers in the context of this model. According to Cook’s distances, none of the studies could be considered to be overly influential. Neither the rank correlation nor the regression test indicated any funnel plot asymmetry (*p* = 1.0000 and *p* = 0.1216, respectively).

**Figure 2:**
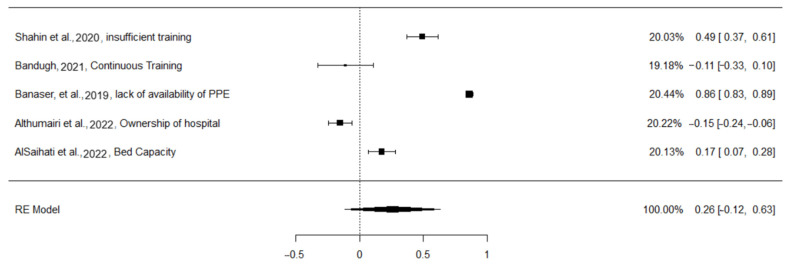
Forest plot of non-compliance factors [29,30,35,36,37].

Figure 2 shows the factors that contribute to non-compliance, which are inadequate training, continuous training, lack of available personal protective equipment (PPE), hospital ownership, and bed capacity. The majority of these factors positively increase the non-compliance, ranging from 0.17 to 0.86. However, the continuous training and ownership of hospitals factors were found to reduce the non-compliance, ranging from −0.11 to −0.15.

## 4. Discussion

The pooled correlations reflected that the factors of non-compliance were minimal, impacting the compliance rate with an overall weak association among the dimensions of healthcare quality. The majority of the studies used the cross-sectional design to assess the factors of non-compliance, with the majority of investigations occurring in hospital settings. This study highlighted that factors fall under the dimensions of safety, efficiency, effectiveness, equity, and patient-centeredness. In comparison, other dimensions were not investigated due to insufficient studies being conducted. The factors varied from the organizational level to the human resources level.

The significant amount of heterogeneity revealed in this analysis implies substantial variation between studies. Potential reasons could be attributed to study design, study variability, population characteristics, and discrepancies in measuring technique. Nonetheless, the geographical location and healthcare system in Saudi Arabia may have an impact on the study outcome and contribute to heterogeneity.

Previous studies found that factors of non-compliance were related to organization staff and resources [39]. Similarly, this study showed that organization and staff were contributing factors for non-compliance. Additionally, despite adequate knowledge levels of infection prevention and control, our study revealed that compliance rates were low. This is inconsistent with previous studies that have found knowledge to be an important part of the compliance rate [40]. Moreover, previous studies have suggested that training is an important factor for compliance. Similarly, this study showed that insufficient training was found to be a major reason for non-compliance [41]. Furthermore, previous systematic reviews indicate that resource accessibility, organization, and physical facilities impact compliance. Similarly, this study showed that organization, equipment availability, and bed capacity can impact non-compliance [42].

The findings of this review illustrate the factors for non-compliance, including training, bed capacity, lack of essential equipment, and hospital ownership, which are aligned with previous studies conducted in the same premises, and found that training was among the factors impacting the compliance rates. However, this study diverges in terms of other factors identified from global trends that involved staff engagement and communication, the hospital’s ability to comply, links to funding mechanisms, and the mandatory versus voluntary nature of accreditation [2,9]. The alignments of the findings regarding training indicate that these factors are universal. However, the divergence of other factors suggests that these factors are contextually based, which may be attributed to the healthcare system and type of accreditation body.

This study provides the key dimensions and areas of factors affecting compliance among healthcare providers through the dimension of healthcare quality. This focus provides a targeted approach to gain a better understanding of non-compliance issues in healthcare. The pooled correlation from multiple studies provides a broader understanding of different study settings and enhances the generalizability of this topic. Based on a foundation of previous studies, this study links non-compliance with several factors such as organization structure, resources, staff, and training, strengthening the argument for addressing these factors to improve compliance rates. This study highlights that compliance may be affected by several factors, indicating a need for further investigation and interventions.

Most of the studies used a cross-sectional design, which could limit the understanding of causality. While this study identifies the factors associated with non-compliance, it may not reflect all factors affecting the non-compliance. Additionally, hospital settings may limit the applicability of the findings to other healthcare institutions. In addition, this study lacks the certain dimension of healthcare quality. The variability in the reported rates could be due to how certain compliance has been measured. Nonetheless, the restriction of English-only articles could lead to potential biases arising from the language restriction. Despite this limitation, this study reported several factors associated with non-compliance and suggested areas for improvement across the dimension of the quality of healthcare. Future research should address the limitations of this study by utilizing more diverse methods and encompassing more variables to investigate the influence of compliance outcomes.

To increase compliance in the context of Saudi Arabia, organizations must improve training programs and evaluate leadership and organizational culture, the availability of resource management, and essential equipment utilization. This study’s practical implications can help accreditation bodies and policymakers to overcome these challenges and find suitable solutions for boosting compliance levels over time by modifying and improving standards and compliance strategies across Saudi Arabian contexts and locations. Nevertheless, by taking care of the factors that have been recognized, such as inadequate training, hospital leaders and managers can target actionable solutions, such as improving training programs and providing the required tools and equipment to improve compliance rates.

## 5. Conclusions

The current study provides insight into the factors affecting non-compliance rates in healthcare settings. This study specifies the dimension of quality in healthcare by pooling correlations from various studies, which highlight a weak association, suggesting that organization, resources, behavior, and perceived non-importance could impact compliance. Moreover, insufficient training is highlighted to be a non-compliance factor. In addition, there is a need to increase the number of studies to investigate the factors of non-compliance, which can improve the overall healthcare delivery within different hospital designs, calling for a more comprehensive approach in future investigations.

The study recommends targeted procedures that can address the identified problems in order to increase compliance rates effectively. Future research should aim to address the identified limitations in this study by using a variety of approaches and investigating a broader range of variables, which will result in better knowledge of the intricate factors of non-compliance in healthcare and will develop more effective measures for improvement.

## Figures and Tables

**Table 1:** Population, intervention, comparison, and outcome (PICO) model.

Items	Terms
Population (P)	Healthcare facilities in Saudi Arabia
Intervention (I)	Accreditation processes
Comparison (C)	Not applicable
Outcome (O)	Factors associated with non-compliance

**Table 3:** Study distribution within the domains.

Dimensions	Reference	N (%)
Safe	[31,32,34,35]	4 (28.6)
Effective	[29,30,32,36]	4 (28.6)
Patient-centered	[33]	1 (7.1)
Timely	No reference	0 (0.0)
Efficient	[29,30,32,36]	4 (28.6)
Equitable	[37]	1 (7.1)
All domains	No reference	0 (0.0)
Total		14 (100)

## Data Availability

The data presented in this study are openly available in [repository name, zenodo.org] at [10.5281/zenodo.14550874], reference number [Compliance]. Registration Information: the review was registered in Open Science Framework (Ref: https://osf.io/25b7d).

## Supplementary Materials

The following supporting information can be downloaded at https://zenodo.org/records/14937977, accessed on 23 February 2025. File S1: PRISMA checklist [43]; File S2: Search strategy; File S3: Risk of bias assessment; File S4: PRESS guidelines.
